# Comparison of Bayesian and frequentist approaches in modelling risk of preterm birth near the Sydney Tar Ponds, Nova Scotia, Canada

**DOI:** 10.1186/1471-2288-7-39

**Published:** 2007-09-10

**Authors:** Afisi S Ismaila, Angelo Canty, Lehana Thabane

**Affiliations:** 1 Department of Clinical Epidemiology and Biostatistics, Faculty of Health Sciences, McMaster University, 1200 Main Street West, Hamilton, ON, L8N 3Z5, Canada; 2 Department of Mathematics and Statistics, McMaster University, 1280 Main Street West, Hamilton, ON, L8S 4K1, Canada; 3 Centre for Evaluation of Medicines, St. Joseph's Healthcare Hamilton, 50 Charlton Avenue East, Room H325, Hamilton, ON L8N 4A6, Canada

## Abstract

**Background:**

This study compares the Bayesian and frequentist (non-Bayesian) approaches in the modelling of the association between the risk of preterm birth and maternal proximity to hazardous waste and pollution from the Sydney Tar Pond site in Nova Scotia, Canada.

**Methods:**

The data includes 1604 observed cases of preterm birth out of a total population of 17559 at risk of preterm birth from 144 enumeration districts in the Cape Breton Regional Municipality. Other covariates include the distance from the Tar Pond; the rate of unemployment to population; the proportion of persons who are separated, divorced or widowed; the proportion of persons who have no high school diploma; the proportion of persons living alone; the proportion of single parent families and average income. Bayesian hierarchical Poisson regression, quasi-likelihood Poisson regression and weighted linear regression models were fitted to the data.

**Results:**

The results of the analyses were compared together with their limitations.

**Conclusion:**

The results of the weighted linear regression and the quasi-likelihood Poisson regression agrees with the result from the Bayesian hierarchical modelling which incorporates the spatial effects.

## Background

Public awareness about potential environmental hazards has continued to grow in recent years. This concern has led to an increased demand for public health authorities and researchers to investigate potential clustering of diseases around putative sources of hazards [1-10]. Evidence of significant association between maternal proximity to hazardous waste sites and risk of low birth-weight and congenital anomalies has been reported in some studies [4-12], but other studies have reported otherwise [1,3,6,13-15]. Some studies have also shown that women exposed to PCB are at increased risk of giving birth to infants with low birth weight [16,17].

An assessment of the effect of human exposure to particular substances can be complex because multiple chemicals are usually involved so it may be difficult to discern the specific agent responsible for a particular health concern [16-19]. Furthermore, extraneous factors, like cultural and socioeconomic, may confound the effect of direct exposure to a waste site [16-24]. Within the boundaries of these limitations, the theory of spatial modelling and its applications to waste landfills and risk of adverse health have been developed and extensively discussed [25-29]. Regression analysis is one of the most widely used methods in the modelling of disease risk associated with proximity to a point source [25]. The parameters of the regression model can be estimated using the Bayesian or the frequentist approaches with spatial data assumed to be available at the individual case level or as spatially aggregated counts in enumeration districts (ED) [25-27].

In this paper, we focus on the comparison of the Bayesian and frequentist regression methods for aggregated counts. Specifically, we compare the Bayesian hierarchical Poisson regression, quasi-likelihood Poisson regression and weighted linear regression modelling approaches in answering the following two questions: 1) Is maternal proximity to hazardous waste and pollution from the Sydney Tar Pond sites associated with increased risk of preterm birth? 2) How much of the variation in preterm birth can be explained by socioeconomic inequalities across the study region?

## Methods

In the following subsections we provide a description of the study site, the data used for analyses and the theoretical framework of methods used to analyse the data.

### Tar Pond site in Sydney

The history of the Tar Pond site in Sydney, Nova Scotia, and the health consequences are well documented [2,30]. The Tar Pond is a tidal estuary of 33 hectares in the Cape Breton regional municipality of Nova Scotia, Canada. This site, considered to be the most toxic site in Canada, is a result of over 100 years of steel manufacturing and other allied industries in the area. The byproducts from these industries include BTEX (benzene, toluene, ethylbenzene, and xylene), PAH (polycyclic aromatic hydrocarbons), PCB (polychlorinated biphenyl) and particulate laden with toxic metals, such as arsenic, lead and other heavy metals. This has led to the contamination of soil and other sources of natural water in the surrounding areas.

### Data description

Cape Breton regional municipality is made up of 158 enumeration districts but aggregated counts of preterm birth were available from only 144 enumeration districts in the municipality. There were 1604 observed cases of preterm birth out of a total population of 17559 at risk of preterm birth. Other variables include the distance from the Tar Pond (*d*) and the following area-specific covariates; the proportion of persons who are separated, divorced or widowed (*x*_2_); the proportion of persons who have no high school diploma (*x*_3_); the proportion of people living alone (*x*_4_); the proportion of single parent families (*x*_5_) and average income (*x*_6_). The covariates were selected based on the Pampalon and Raymond index [21] for health and welfare planning in Quebec. All area-specific covariates were extracted from the 1996 Canadian census data.

### Some theoretical background and context

Let *Y*_*i *_denote the number of observed cases of preterm birth, and *N*_*i *_the population at risk in each enumeration district (ED). The expected counts (*E*_*i*_) for each ED was calculated by multiplying *N*_*i *_by the the Canada preterm birth rate of 7.1 per 100 live births in 1996 (source: Population and Public Health Branch, Health Canada). This rate is assumed fixed for 1996 and may have been calculated by including data from the Cape Breton regional municipality, but we will assume that the effect of this can be ignored. Hence, *E*_*i *_is the expected number of preterm birth from all other sources of risk other than pollution from the Sydney Tar Pond. Preterm births only occur in females within the child-bearing age and the condition is not infectious. Hence, it is reasonable to assume that each case occurred independently. We also assumed that the risk is constant in each ED, so that

*Y*_*i*_|*λ*_*i *_~ Poisson (*E*_*i*_*λ*_*i*_) *i *= 1,..., *n*,

where *λ*_*i *_denotes the relative risk of preterm birth for each ED compared to the whole country [31]. The maximum likelihood estimator of *λ*_*i *_is the unadjusted standardized incidence ratio (SIR), the ratio of observed to expected within each ED [27,32]. We use a regression approach to adjust the crude SIR to improve its stability where the population at risk may be small [27,29,32,33].

Based on the work of Morris and Wakefield [27], we define the null hypothesis that proximity to source does not influence risk by

*H*_0 _: *λ*_*i *_= *η *for *i *= 1,..., *n*.

Now suppose (*x*_0_, *y*_0_) denotes the centroid of the Tar pond, (*x*_*i*_, *y*_*i*_) the centroid of each ED and *d*_*i *_the distance between the two centroid. In the absence of an exposure measure that may be attached to each ED, Morris and Wakefield [27] define a natural additive distance/risk model by

*λ*_*i *_= *η *{1 + *f*(*d*_*i*_; *θ*)}

where *η *is the background relative risk and *f*(*d*_*i*_; *θ*) is a function of distance, such that *f *(*d*_*i*_; *θ*) → 0 as *d*_*i *_→ ∞. We will use a reparameterization of the form

*λ*_*i *_= *η g*(*d*_*i*_; *θ*)

so that this model will be consistent with Bithell [34]. With this reparameterization, *g*(*d*_*i*_; *θ*) → 1 as *d*_*i *_→ ∞. Bithell [34] proposed the following distance functions as suitable forms for *g*(*d*_*i*_).

*g*_1_(*d*_*i*_) = exp(*α*/*d*_*i*_)

*g*_2_(*d*_*i*_) = 1 + *ξ *exp(-*d*_*i*_/*β*)

*g*_3_(*d*_*i*_) = 1 + *ξ *exp(-(*d*_*i*_/*γ*)^2^)

*g*_4_(*d*_*i*_) = 1 + *ξ*/(1 + *d*_*i*_/*δ*)

where *α*, *β*, *γ*, and *δ *represent decay rates. For *g*_2_(*d*_*i*_), *g*_3_(*d*_*i*_) and *g*_4_(*d*_*i*_), 1 + *ξ *is a measure of the ratio of relative risk at source to that at infinity. Other variants of the Bithell functions have also been proposed [35]. For simplicity, and following Datta *et al. *[32] and Bithell [34], we have chosen

*g*(*d*_*i*_; *θ*) = *g*_1 _(*d*_*i*_; *θ*) = exp(*α*/*d*_*i*_).

We incorporated the area-level covariates (*z*_*i*_) and a measure of the spread of the risk from the Tar pond through a generalized linear model of the form

log⁡λi=αo+log⁡g1(di;θ)+ziTφ=αo+αdi+ziTφ
 MathType@MTEF@5@5@+=feaafiart1ev1aaatCvAUfKttLearuWrP9MDH5MBPbIqV92AaeXatLxBI9gBaebbnrfifHhDYfgasaacH8akY=wiFfYdH8Gipec8Eeeu0xXdbba9frFj0=OqFfea0dXdd9vqai=hGuQ8kuc9pgc9s8qqaq=dirpe0xb9q8qiLsFr0=vr0=vr0dc8meaabaqaciaacaGaaeqabaqabeGadaaakeaacyGGSbaBcqGGVbWBcqGGNbWziiGacqWF7oaBdaWgaaWcbaGaemyAaKgabeaakiabg2da9iab=f7aHnaaBaaaleaacqWGVbWBaeqaaOGaey4kaSIagiiBaWMaei4Ba8Maei4zaCMaem4zaC2aaSbaaSqaaiabigdaXaqabaGccqGGOaakcqWGKbazdaWgaaWcbaGaemyAaKgabeaakiabcUda7iab=H7aXjabcMcaPiabgUcaRiabdQha6naaDaaaleaacqWGPbqAaeaacqWGubavaaGccqWFgpGzcqGH9aqpcqWFXoqydaWgaaWcbaGaem4Ba8gabeaakiabgUcaRmaalaaabaGae8xSdegabaGaemizaq2aaSbaaSqaaiabdMgaPbqabaaaaOGaey4kaSIaemOEaO3aa0baaSqaaiabdMgaPbqaaiabdsfaubaakiab=z8aMbaa@5E6A@

where *α*_*o *_= log *η*. Hence, *η *= exp(*α*_*o*_) is a measure of the overall inflation of risk in the region under study, *α *represents the decay rate and *ϕ *is a vector of parameters of the area-specific covariates. One of the problems associated with the use of equation (7) is overdispersion (heterogeneity or spatial dependency) [36]. In the frequentist framework, we have assessed spatial autocorrelation by using any of the Moran's I statistics [37]. Other alternatives include Geary's C statistic [38] and non-parametric rank-based method [39]. The Bayesian approach is discussed in the next section.

### Bayesian hierarchical modelling

To model the data while accommodating the expected heterogeneity and also including the spatial components (location or relative position of data values) of the data, Bayesian hierarchical modelling [33,40,41] was used. The implementation of this modelling was done with WINBUGS and GeoBugs software [42] for modelling aggregated data with plots and convergence diagnostic tests done using the **coda **package in R [43]. The mean or median of the posterior distribution is used as a point estimate of disease risk for each area. The modelling is explained in the following three stages:

#### First-stage: model

We incorporated two measures of overdispersion, so that equation (7) becomes

log⁡λi=αo+α/di+ziTφ+Vi+Ui
 MathType@MTEF@5@5@+=feaafiart1ev1aaatCvAUfKttLearuWrP9MDH5MBPbIqV92AaeXatLxBI9gBaebbnrfifHhDYfgasaacH8akY=wiFfYdH8Gipec8Eeeu0xXdbba9frFj0=OqFfea0dXdd9vqai=hGuQ8kuc9pgc9s8qqaq=dirpe0xb9q8qiLsFr0=vr0=vr0dc8meaabaqaciaacaGaaeqabaqabeGadaaakeaacyGGSbaBcqGGVbWBcqGGNbWziiGacqWF7oaBdaWgaaWcbaGaemyAaKgabeaakiabg2da9iab=f7aHnaaBaaaleaacqWGVbWBaeqaaOGaey4kaSIae8xSdeMaei4la8Iaemizaq2aaSbaaSqaaiabdMgaPbqabaGccqGHRaWkcqWG6bGEdaqhaaWcbaGaemyAaKgabaGaemivaqfaaOGae8NXdyMaey4kaSIaemOvay1aaSbaaSqaaiabdMgaPbqabaGccqGHRaWkcqWGvbqvdaWgaaWcbaGaemyAaKgabeaaaaa@4CB1@

where *V*_*i *_are unstructured random effects included in the model to capture the effects of unknown or unmeasured area level covariates. Hence, exp(*V*_*i*_) will be equal to the residual or unexplained relative risk in each ED after adjusting for known area-specific covariates. We have included *U*_*i *_in the model to capture our belief that the unstructured random effects (*V*_*i*_) may exhibit some spatial structure.

#### Second-stage: overdispersion modelling

We assume that the unstructured random effects which is a measure of heterogeneity is of the form

Vi~iidN(0,σv2)i=1,...,n
 MathType@MTEF@5@5@+=feaafiart1ev1aaatCvAUfKttLearuWrP9MDH5MBPbIqV92AaeXatLxBI9gBaebbnrfifHhDYfgasaacH8akY=wiFfYdH8Gipec8Eeeu0xXdbba9frFj0=OqFfea0dXdd9vqai=hGuQ8kuc9pgc9s8qqaq=dirpe0xb9q8qiLsFr0=vr0=vr0dc8meaabaqaciaacaGaaeqabaqabeGadaaakeaafaqabeqacaaabaGaemOvay1aaSbaaSqaaiabdMgaPbqabaGcdaWfGaqaamaaxababaGaeiOFa4haleaaaeqaaaqabeaacqWGPbqAcqWGPbqAcqWGKbazaaGccqWGobGtcqGGOaakcqaIWaamcqGGSaaliiGacqWFdpWCdaqhaaWcbaGaemODayhabaGaeGOmaidaaOGaeiykaKcabaGaemyAaKMaeyypa0JaeGymaeJaeiilaWIaeiOla4IaeiOla4IaeiOla4IaeiilaWIaemOBa4gaaaaa@47BA@

where σv2
 MathType@MTEF@5@5@+=feaafiart1ev1aaatCvAUfKttLearuWrP9MDH5MBPbIqV92AaeXatLxBI9gBaebbnrfifHhDYfgasaacH8akY=wiFfYdH8Gipec8Eeeu0xXdbba9frFj0=OqFfea0dXdd9vqai=hGuQ8kuc9pgc9s8qqaq=dirpe0xb9q8qiLsFr0=vr0=vr0dc8meaabaqaciaacaGaaeqabaqabeGadaaakeaaiiGacqWFdpWCdaqhaaWcbaGaemODayhabaGaeGOmaidaaaaa@310A@ is a measure of the between-area variability of the *V*_*i*_. Next, we specify the spatial random effect to model the anticipated spatial dependence of the log of relative risk. For a detailed review on the modelling of the spatial variability see Wakefield *et al. *[26,41].

We specified the Markov random field (MRF) model using the intrinsic conditional autoregressive (CAR) proposed by Besag *et al. *[40]. We define ED *i *and *j *as neighbours if they share a common boundary [31,40,41]. We also define the spatial weights {*W*_*ij *_: *i *= 1,..., *n*} as a binary contiguity matrix in which *W*_*ij *_= 1 for neighbours and *W*_*ij *_= 0 otherwise. Furthermore, *W*_*ii *_= 0 and the constraint ∑i=1nUi=0
 MathType@MTEF@5@5@+=feaafiart1ev1aaatCvAUfKttLearuWrP9MDH5MBPbIqV92AaeXatLxBI9gBaebbnrfifHhDYfgasaacH8akY=wiFfYdH8Gipec8Eeeu0xXdbba9frFj0=OqFfea0dXdd9vqai=hGuQ8kuc9pgc9s8qqaq=dirpe0xb9q8qiLsFr0=vr0=vr0dc8meaabaqaciaacaGaaeqabaqabeGadaaakeaadaaeWaqaaiabdwfavnaaBaaaleaacqWGPbqAaeqaaOGaeyypa0JaeGimaadaleaacqWGPbqAcqGH9aqpcqaIXaqmaeaacqWGUbGBa0GaeyyeIuoaaaa@381C@ is imposed for identifiability.

#### Third-stage: prior distributions

At this stage all the parameters (*α*_*o*_, *α*, *ϕ*, σv−2
 MathType@MTEF@5@5@+=feaafiart1ev1aaatCvAUfKttLearuWrP9MDH5MBPbIqV92AaeXatLxBI9gBaebbnrfifHhDYfgasaacH8akY=wiFfYdH8Gipec8Eeeu0xXdbba9frFj0=OqFfea0dXdd9vqai=hGuQ8kuc9pgc9s8qqaq=dirpe0xb9q8qiLsFr0=vr0=vr0dc8meaabaqaciaacaGaaeqabaqabeGadaaakeaaiiGacqWFdpWCdaqhaaWcbaGaemODayhabaGaeyOeI0IaeGOmaidaaaaa@31F7@ and σu−2
 MathType@MTEF@5@5@+=feaafiart1ev1aaatCvAUfKttLearuWrP9MDH5MBPbIqV92AaeXatLxBI9gBaebbnrfifHhDYfgasaacH8akY=wiFfYdH8Gipec8Eeeu0xXdbba9frFj0=OqFfea0dXdd9vqai=hGuQ8kuc9pgc9s8qqaq=dirpe0xb9q8qiLsFr0=vr0=vr0dc8meaabaqaciaacaGaaeqabaqabeGadaaakeaaiiGacqWFdpWCdaqhaaWcbaGaemyDauhabaGaeyOeI0IaeGOmaidaaaaa@31F5@) of the model are assigned a prior distribution. *α*_*o *_was assigned a flat prior which corresponds to a uniform distribution over the whole real line. *α*, and *ϕ*_*i*_were assigned a normal (0, 10^5^). The choice of prior for σv−2
 MathType@MTEF@5@5@+=feaafiart1ev1aaatCvAUfKttLearuWrP9MDH5MBPbIqV92AaeXatLxBI9gBaebbnrfifHhDYfgasaacH8akY=wiFfYdH8Gipec8Eeeu0xXdbba9frFj0=OqFfea0dXdd9vqai=hGuQ8kuc9pgc9s8qqaq=dirpe0xb9q8qiLsFr0=vr0=vr0dc8meaabaqaciaacaGaaeqabaqabeGadaaakeaaiiGacqWFdpWCdaqhaaWcbaGaemODayhabaGaeyOeI0IaeGOmaidaaaaa@31F7@ and σu−2
 MathType@MTEF@5@5@+=feaafiart1ev1aaatCvAUfKttLearuWrP9MDH5MBPbIqV92AaeXatLxBI9gBaebbnrfifHhDYfgasaacH8akY=wiFfYdH8Gipec8Eeeu0xXdbba9frFj0=OqFfea0dXdd9vqai=hGuQ8kuc9pgc9s8qqaq=dirpe0xb9q8qiLsFr0=vr0=vr0dc8meaabaqaciaacaGaaeqabaqabeGadaaakeaaiiGacqWFdpWCdaqhaaWcbaGaemyDauhabaGaeyOeI0IaeGOmaidaaaaa@31F5@ is a very challenging one and it has to be done carefully. Many authors have favoured the use of gamma (a, b) for both σv−2
 MathType@MTEF@5@5@+=feaafiart1ev1aaatCvAUfKttLearuWrP9MDH5MBPbIqV92AaeXatLxBI9gBaebbnrfifHhDYfgasaacH8akY=wiFfYdH8Gipec8Eeeu0xXdbba9frFj0=OqFfea0dXdd9vqai=hGuQ8kuc9pgc9s8qqaq=dirpe0xb9q8qiLsFr0=vr0=vr0dc8meaabaqaciaacaGaaeqabaqabeGadaaakeaaiiGacqWFdpWCdaqhaaWcbaGaemODayhabaGaeyOeI0IaeGOmaidaaaaa@31F7@ and σu−2
 MathType@MTEF@5@5@+=feaafiart1ev1aaatCvAUfKttLearuWrP9MDH5MBPbIqV92AaeXatLxBI9gBaebbnrfifHhDYfgasaacH8akY=wiFfYdH8Gipec8Eeeu0xXdbba9frFj0=OqFfea0dXdd9vqai=hGuQ8kuc9pgc9s8qqaq=dirpe0xb9q8qiLsFr0=vr0=vr0dc8meaabaqaciaacaGaaeqabaqabeGadaaakeaaiiGacqWFdpWCdaqhaaWcbaGaemyDauhabaGaeyOeI0IaeGOmaidaaaaa@31F5@ because it is a conjugate prior in the normal model but the choice of *a *and *b *is what they have not agreed on [31-33,36,40,41]. In our case, we have assigned gamma (0.1, 0.1) to both σv−2
 MathType@MTEF@5@5@+=feaafiart1ev1aaatCvAUfKttLearuWrP9MDH5MBPbIqV92AaeXatLxBI9gBaebbnrfifHhDYfgasaacH8akY=wiFfYdH8Gipec8Eeeu0xXdbba9frFj0=OqFfea0dXdd9vqai=hGuQ8kuc9pgc9s8qqaq=dirpe0xb9q8qiLsFr0=vr0=vr0dc8meaabaqaciaacaGaaeqabaqabeGadaaakeaaiiGacqWFdpWCdaqhaaWcbaGaemODayhabaGaeyOeI0IaeGOmaidaaaaa@31F7@ and σu−2
 MathType@MTEF@5@5@+=feaafiart1ev1aaatCvAUfKttLearuWrP9MDH5MBPbIqV92AaeXatLxBI9gBaebbnrfifHhDYfgasaacH8akY=wiFfYdH8Gipec8Eeeu0xXdbba9frFj0=OqFfea0dXdd9vqai=hGuQ8kuc9pgc9s8qqaq=dirpe0xb9q8qiLsFr0=vr0=vr0dc8meaabaqaciaacaGaaeqabaqabeGadaaakeaaiiGacqWFdpWCdaqhaaWcbaGaemyDauhabaGaeyOeI0IaeGOmaidaaaaa@31F5@ and carry out sensitivity analysis with all the priors given in [31-33,36,40,41].

The models were fitted using Markov Chain Monte Carlo (MCMC) simulation method [44]. Five separate chains starting from different initial values were run for each model. Convergence was assessed by visual examination of time series plots for each parameter and by carrying out the Gelman and Rubin diagnostic test [45] based on the ratio of between to within chain variances for each model. The time series plots with all the five chains superimposed were examined to see whether the chains were mixing well. Goodness of fit was examined using the Deviance Information Criterion (DIC) [46] which consists of two terms, one is a measure of goodness of fit and the other is a penalty for increasing model complexity so that smaller values of DIC indicate a better-fitting model.

We defined a quantity *ψ *= *σ*_*u*_/(*σ*_*u *_+ *σ*_*v*_) as a measure of the relative contribution of *U*_*i *_and *V*_*i *_to the total overdispersion [33]. So that as *ψ *→ 1, spatial variation dominates, while as *ψ *→ 0, spatial variation becomes negligible.

### Poisson regression

For *Y*_*i *_~ Poisson(*μ*_*i*_), where *μ*_*i *_= *λ*_*i*_E_*i *_(*i *= 1,..., *n*), we assume the generalized linear model [47]. Four models were fitted for the log relative risk (log *λ*_*i *_= log *μ*_*i *_- log *E*_*i*_) in terms of a constant, area-level covariates and the reciprocal of distance. The fitted models are:

log *λ*_*i *_= *α*_*o*_

log *λ*_*i *_= *α*_*o *_+ *α*/*d*_*i*_

log *λ*_*i *_= *α*_*o *_+ *ϕ*_1_*x*_1 _+ *ϕ*_2_*x*_2 _+ *ϕ*_3_*x*_3 _+ *ϕ*_4_*x*_4 _+ *ϕ*_5_*x*_5_

log *λ*_*i *_= *α*_*o *_+ *α*/*d*_*i *_+ *ϕ*_1_*x*_1 _+ *ϕ*_2_*x*_2 _+ *ϕ*_3_*x*_3 _+ *ϕ*_4_*x*_4 _+ *ϕ*_5_*x*_5_

No random effects or spatial effects was included. In each of the fitted models, log *E*_*i *_is used as an offset to account for variations in *λ*_*i *_over the study region. The models were fitted using the quasi-likelihood approach to account for the overdispersion that might occur in the data set. The dispersion parameter, *κ*, was estimated by the mean of the Pearson *χ*^2 ^statistic.

### Weighted linear regression

A weighted regression approach was carried out to account for the dispersion that might result from the violation of the constant variance assumption in the least squares approach. The last three models (equations 10, 11 and 12) were fitted using weighted least square regression by replacing *λ*_*i *_with the SIR (λ^i
 MathType@MTEF@5@5@+=feaafiart1ev1aaatCvAUfKttLearuWrP9MDH5MBPbIqV92AaeXatLxBI9gBaebbnrfifHhDYfgasaacH8akY=wiFfYdH8Gipec8Eeeu0xXdbba9frFj0=OqFfea0dXdd9vqai=hGuQ8kuc9pgc9s8qqaq=dirpe0xb9q8qiLsFr0=vr0=vr0dc8meaabaqaciaacaGaaeqabaqabeGadaaakeaaiiGacuWF7oaBgaqcamaaBaaaleaacqWGPbqAaeqaaaaa@2FFE@ = *Y*_*i*_*/E*_*i*_). The weights (*w*_*i*_) were set equal to Ei/∑i=1nEi
 MathType@MTEF@5@5@+=feaafiart1ev1aaatCvAUfKttLearuWrP9MDH5MBPbIqV92AaeXatLxBI9gBaebbnrfifHhDYfgasaacH8akY=wiFfYdH8Gipec8Eeeu0xXdbba9frFj0=OqFfea0dXdd9vqai=hGuQ8kuc9pgc9s8qqaq=dirpe0xb9q8qiLsFr0=vr0=vr0dc8meaabaqaciaacaGaaeqabaqabeGadaaakeaacqWGfbqrdaWgaaWcbaGaemyAaKgabeaakiabc+caVmaaqadabaGaemyrau0aaSbaaSqaaiabdMgaPbqabaaabaGaemyAaKMaeyypa0JaeGymaedabaGaemOBa4ganiabggHiLdaaaa@397D@ so that the error sum of squares (*Q*) of the weighted linear regression can be written as

Q=∑1=1nwi{log⁡λ^i−(αo+α/di+φ1x1+φ2x2+φ3x3+φ4x4+φ5x5)}2.
 MathType@MTEF@5@5@+=feaafiart1ev1aaatCvAUfKttLearuWrP9MDH5MBPbIqV92AaeXatLxBI9gBaebbnrfifHhDYfgasaacH8akY=wiFfYdH8Gipec8Eeeu0xXdbba9frFj0=OqFfea0dXdd9vqai=hGuQ8kuc9pgc9s8qqaq=dirpe0xb9q8qiLsFr0=vr0=vr0dc8meaabaqaciaacaGaaeqabaqabeGadaaakeaacqWGrbqucqGH9aqpdaaeWbqaaiabdEha3naaBaaaleaacqWGPbqAaeqaaOGaei4EaSNagiiBaWMaei4Ba8Maei4zaCgcciGaf83UdWMbaKaadaWgaaWcbaGaemyAaKgabeaakiabgkHiTiabcIcaOiab=f7aHnaaBaaaleaacqWGVbWBaeqaaOGaey4kaSIae8xSdeMaei4la8Iaemizaq2aaSbaaSqaaiabdMgaPbqabaGccqGHRaWkcqWFgpGzdaWgaaWcbaGaeGymaedabeaakiabdIha4naaBaaaleaacqaIXaqmaeqaaOGaey4kaSIae8NXdy2aaSbaaSqaaiabikdaYaqabaGccqWG4baEdaWgaaWcbaGaeGOmaidabeaakiabgUcaRiab=z8aMnaaBaaaleaacqaIZaWmaeqaaOGaemiEaG3aaSbaaSqaaiabiodaZaqabaGccqGHRaWkcqWFgpGzdaWgaaWcbaGaeGinaqdabeaakiabdIha4naaBaaaleaacqaI0aanaeqaaOGaey4kaSIae8NXdy2aaSbaaSqaaiabiwda1aqabaGccqWG4baEdaWgaaWcbaGaeGynaudabeaakiabcMcaPiabc2ha9naaCaaaleqabaGaeGOmaidaaaqaaiabigdaXiabg2da9iabigdaXaqaaiabd6gaUbqdcqGHris5aOGaeiOla4caaa@70F7@

Here, we have not included the spatial component of the model because we have seen that the SIR does not exhibit spatial dependency during our exploratory data analysis.

## Results

In the following subsections, we explain the results of the exploratory data analysis and modelling.

### Exploratory data analysis

**Figure 1:**
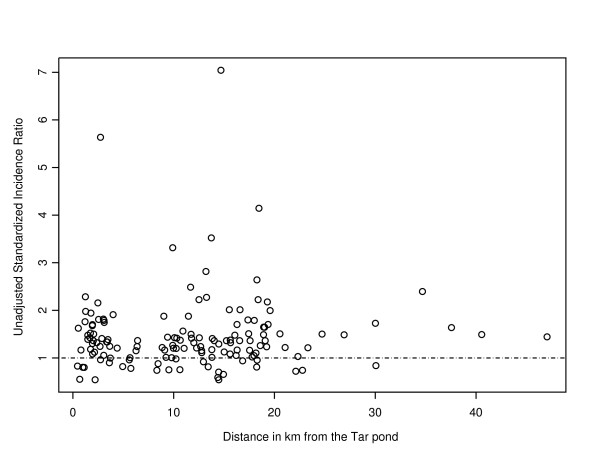
**Plot of SIR against distance**. Plot of unadjusted standardized incidence ratios against distance in km from the Tar Pond.

**Figure 2:**
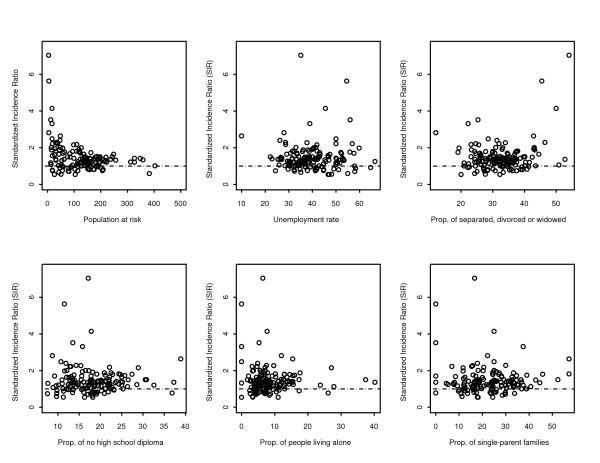
**Plot of SIR**. Plot of SIR versus population at risk and other area-specific covariates.

Plot of unadjusted standardized incidence ratios (SIR) against distance in km from the Tar Pond is shown in Figure 1. From the plots, areas with SIR less than 1 indicate no risk or absolute risk reduction while SIR greater than 1 indicate high risk of preterm birth compared to the rest of Canada. All the high values of SIR occurred within the 20 km distance from the Tar Pond. There is some evidence of decrease in risk from source as we move further away but this will be tested statistically in the next sections. However, as explained earlier, care has to be taken when interpreting the crude SIR. To illustrate this, we plotted the SIR against the population at risk (see Figure 2). This graph clearly shows that areas with low population at risk tend to show high variability in SIR. We accounted for this by using the Poisson model regression for aggregated data.

### Area-specific risk

Following Pampalon and Raymond [21], the following area-specific variables were considered for the analysis: the proportion of persons who have no high school diploma, the rate of unemployment, average income, the proportion of persons who are separated, divorced or widowed, the proportion of single parent families and the proportion of people living alone.

Only five of the variables are available at all the 144 EDs with average income available only in 130 EDs. Hence, we could not compute an adequate measure of deprivation based on the method proposed by Pampalon and Raymond. We decided to assess the effect of each of the variables separately leaving out average income. Distance from the Tar Pond site and all the area-specific variables were plotted against SIR to assess the effect of each. The plots are given in Figure 2.

As explained earlier, points below the dotted line indicate no risk or absolute risk reduction and vice versa. The plot of SIR and the rate of unemployment shows an upward trend with high unemployment rates associated with high SIR. A similar pattern is displayed by the plot of SIR against proportion of persons with no high school diploma. In the plot of the SIR against proportion of separated, divorced and widowed areas with low proportion of separated, divorced and widowed tend to have high SIR. A similar pattern is seen in the plot of SIR and proportion of people living alone. There is no obvious pattern in the plot of SIR against proportion of single parent families.

### Test for spatial dependency

**Figure 3:**
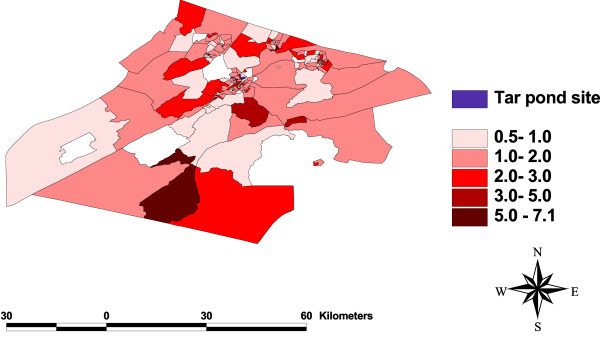
**Map of SIR**. A map showing the unadjusted standardized incidence ratios for preterm birth.

**Figure 4:**
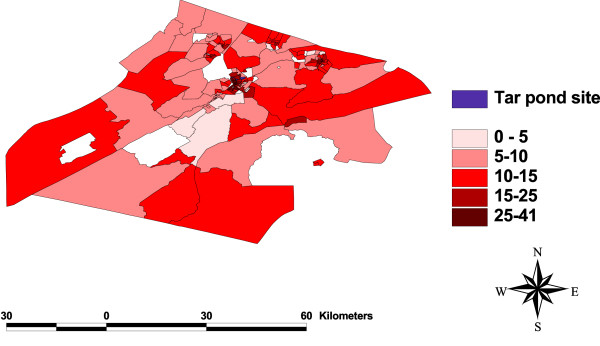
**Map of people living alone**. A map showing the percentage of people living alone.

**Figure 5:**
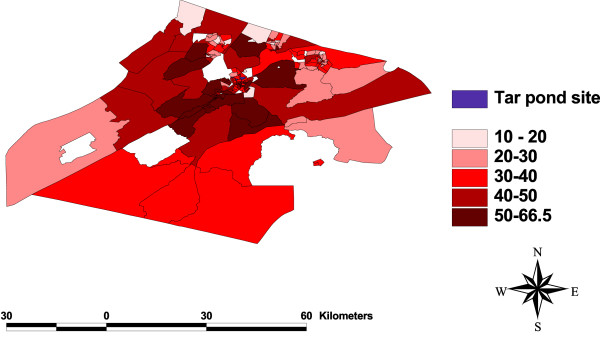
**Map of rate of unemployment**. A map showing the rate of unemployment to population.

**Figure 6:**
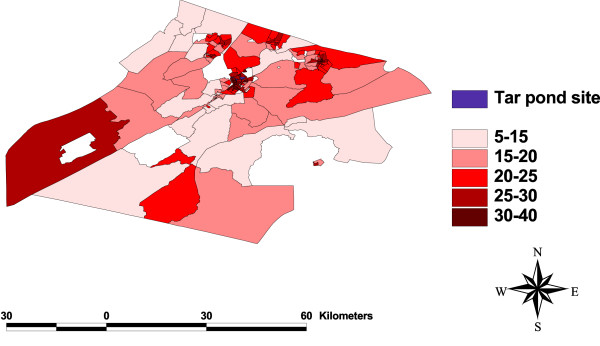
**Map of persons separated, divorced or widowed**. A map showing the percentage of persons who are separated, divorced or widowed.

**Figure 7:**
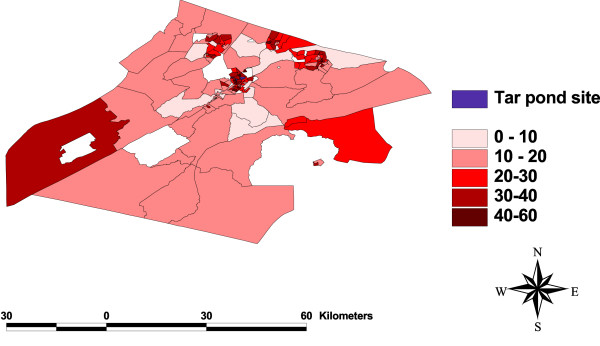
**Map of single parent families**. A map showing the percentage of single parent families.

**Figure 8:**
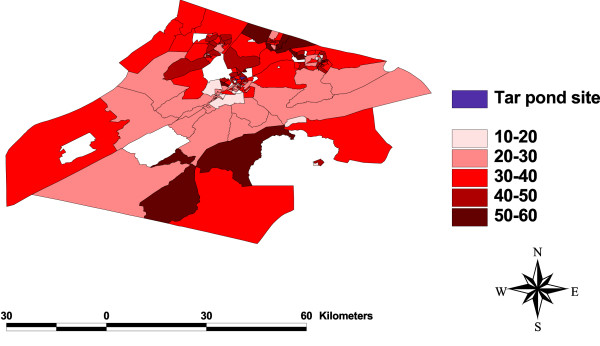
**Map of persons who have no high school diploma**. A map showing the percentage of persons who have no high school diploma.

One of the objectives of this study is to check for any clustering of events around the Tar Pond that may be significant in explaining the variation in preterm birth rates. This was done by plotting the maps of all the variables (Figure 3, 4, 5, 6, 7, 8) and visually assessing whether there is any clustering, and by performing a formal test of clustering using the Moran I statistic [37].

The map of SIR (Figure 3) was examined to see whether there is a cluster of high SIR around the Tar Pond or a decrease in the SIR as we move further away from the Tar Pond but neither of the two is obvious from the map. The maps of all the area-covariates (Figure 4, 5, 6, 7, 8) were examined to assess whether there is any spatial pattern. The plot of percentage of people living alone shows a pattern with the highest proportion of people living alone occurring within the 20 km radius of the Tar Pond site. The plot of the rate of unemployment to population also shows that the unemployment to population ratio decreases as we move further away from the Tar Pond. Furthermore, the proportion of persons who have no high school diploma also displays some spatial pattern with some of the areas close to the Tar Pond having high proportion. Existence of spatial autocorrelation was also tested formally using the Moran I test. Results of the spatial autocorrelation analysis given in Table 1, show the correlation, standard error, corresponding normal statistic and associated *p*-value. The results indicate that there is significant autocorrelation for all the covariates with the exception of SIR (*p *= 0.5387). However, a more confirmatory test is required. The result of the autocorrelation not being statistically significant could be due to different reasons (1) there is none; or (2) small populations which may give rise to high SIR [39].

**Table 1:** Results of spatial autocorrelation analysis using Moran I statistics

Variables	Correlation	Std. Error	Normal statistic	Normal *p*-value
SIR	-0.03798	0.05041	-0.6148	0.5387
*x*_1_	0.348	0.05041	7.043	*p *< 0.0001
*x*_2_	0.4582	0.05041	9.229	*p *< 0.0001
*x*_3_	0.1924	0.05041	3.955	*p *< 0.0001
*x*_4_	0.4051	0.05041	8.174	*p *< 0.0001
*x*_5_	0.2932	0.05041	5.955	*p *< 0.0001

### Bayesian analysis results

The following four models were fitted using the five area covariates available at all the 144 EDs and a measure of proximity (*d*_*i*_): Model 1 which contains no covariates and corresponds to the null model; Model 2 which contains the distance measure alone; Model 3 with deprivation covariates alone; and finally, Model 4 with distance and deprivation covariates.

The Gelman Rubin Plots shows that the "shrinkage factor" for each parameter approaches 1. Hence, all chains have escaped the influence of their starting points. The autocorrelation plots shows that autocorrelation decrease rapidly from lag 1. On this basis, the first 2000 samples of each chain were discarded as 'burn-in'; each chain was run for a further 10,000 iterations, and posterior estimates were based on pooling the 5 × 10, 000 samples for each model. This gave Monte Carlo standard errors that are less than 1% of the posterior standard deviation for each parameter. All the plots including the posterior density of each parameter after convergence are provided as additional file 1 (Bayesian diagnostic  plots). All the plots were produced with the **coda **package for R [43].

Table 2 gives the summaries of the posterior distribution under each model. From Table 2, we can see that estimates of *α *in both Models 2 and 4 are negative, and the 95% credible intervals contain zero which shows that there is no significant association between distance from the Tar Ponds and risk of preterm birth. The 95% credible interval for *ϕ*_*i *_(*i *= 1,..., 5) in Models 3 and 4 also contain zero which shows that the change in risk cannot be explained by any of the socio-economic covariates. For each of the models, *η*, a measure of the overall risk, was found to be greater than 1 which is evidence that there is an increased risk of preterm birth in the entire Cape Breton region compared to the rest of Canada.

**Table 2:** Bayesian posterior median (95% credible interval), summaries of model fit (DIC) and complexity (*p*_*D*_)

Nodes	Model 1	Model 2	Model 3	Model 4
*α*	-	-0.097 (-0.326,0.120)	-	-0.087 (-0.317,0.130)
*α*_*o*_	0.246 (0.188,0.305)	0.268 (0.193,0.343)	0.241 (0.182,0.300)	0.260 (0.183,0.336)
*ϕ*_1_	-	-	-0.019 (-0.108,0.070)	-0.019 (-0.107,0.072)
*ϕ*_2_	-	-	-0.001 (-0.080,0.077)	0.001 (-0.079,0.080)
*ϕ*_3_	-	-	0.051 (-0.091,0.195)	0.049 (-0.092,0.189)
*ϕ*_4_	-	-	0.008 (-0.102,0.118)	0.008 (-0.101,0.116)
*ϕ*_5_	-	-	-0.002 (-0.093,0.090)	-0.002 (-0.092,0.090)
*ψ*	0.557 (0.428,0.676)	0.559 (0.434,0.679)	0.555 (0.426,0.677)	0.558 (0.430,0.682)
*η*	1.279 (1.207,1.356)	1.307 (1.212,1.409)	1.272 (1.200,1.349)	1.297 (1.201,1.400)
*σ*_*u*_	0.187 (0.125,0.281)	0.189 (0.127,0.283)	0.185 (0.124,0.282)	0.187 (0.126,0.287)
*σ*_*v*_	0.149 (0.109,0.204)	0.149 (0.110,0.204)	0.149 (0.108,0.204)	0.149 (0.108,0.203)
DIC	727.934	728.672	732.164	734.653
*p*_*D*_	38.419	39.208	42.915	41.094

**Figure 9:**
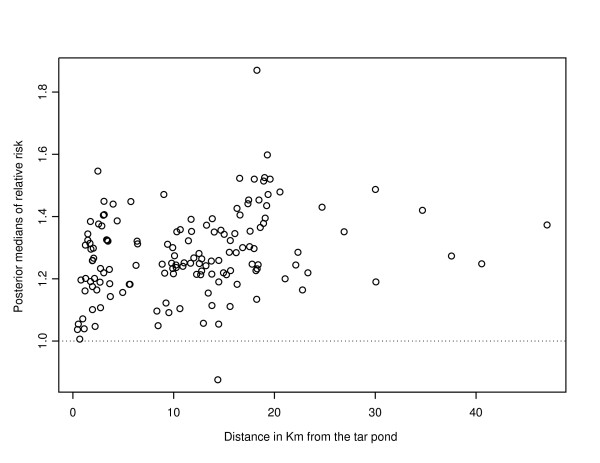
**Plot of posterior medians**. Plot of the posterior medians of relative risk against distance from Tar Pond in km.

The parameters, *σ*_*u *_and *σ*_*v *_change only slightly over the four models. From Table 2, the 95% credible intervals for *ψ *for each model contain 0.5. Hence, there is no clear evidence that the spatial structure dominates the random effect in any of the models. From the results of Table 2, the DIC increases as more variables are added into the model. Hence, Model 1 is better than all three other models. Finally, the posterior median of the relative risk of preterm birth were plotted against distance in km from the Tar pond. The plot is shown in Figure 9.

**Figure 10:**
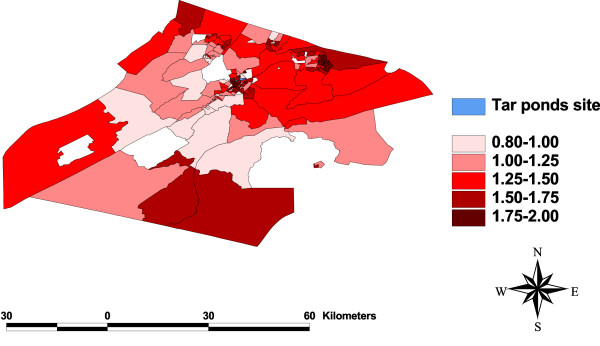
**Map of posterior medians**. A map showing the posterior median of the relative risk of preterm births for Model 4.

A comparison of the plots with that of Figure 1 shows there is a high relative risk (greater than 1) of preterm birth in almost all the enumeration districts. However, the risk is not as high in Figure 9 as in Figure 1. The plots also show that there is no clear relationship between distance and risk. This result is consistent with the results of two of the studies conducted in this area using primary data [1,3]. They both concluded that a causal association between preterm births and maternal/residential proximity to the Tar Ponds could not be inferred from the statistical analysis. A map showing the posterior median of the relative risk of preterm births for Model 4 is shown in Figure 10.

### Poisson regression analysis results

The results of all four models are displayed in Table 3. For each of the fitted models, *κ *was estimated to be approximately equal to 1, a condition that shows that there is no evidence of overdispersion. The Wald confidence intervals shown in Table 3 are based on the asymptotic normality of the parameter estimators. From the table, we can see that the estimated *α *in both Models 2 and 4 is negative, and the 95% Wald confidence intervals contain zero which is evidence that there is no decrease in risk as distance from Tar pond decreases.

**Table 3:** Poisson regression parameter estimates (95% Wald CI), residual deviance and over-dispersion parameter

Parameter	Model 1	Model 2	Model 3	Model 4
*α*	-	-0.0878(-0.2519,0.0763)	-	-0.075 (-0.239,0.089)
*α*_*o*_	0.2520	0.2707 (0.2111,0.3303)	0.2163 (-0.3427,0.7753)	0.226 (-0.334,0.785)
*ϕ*_1_	-	-	-0.0034 (-0.0103,0.0035)	-0.003 (-0.010,0.004)
*ϕ*_2_	-	-	-0.0008 (-0.0099,0.0083)	-0.0005 (-0.0096,0.0086)
*ϕ*_3_	-	-	0.0115 (-0.0074,0.0305)	0.011 (-0.008,0.030)
*ϕ*_4_	-	-	0.0007 (-0.0128,0.0142)	0.0006 (-0.0129,0.0141)
*ϕ*_5_	-	-	-0.0011 (-0.0079,0.0057)	-0.0010 (-0.0078,0.0058)
*η*	1.287	1.311(1.235,1.391)	1.241(0.710,2.171)	1.254(0.716,2.192)
Deviance	132	130.56	122.9983	122.18
Df	143	142	138	137
*κ*	0.99	0.9942	0.9887	0.9906

The 95% confidence intervals for *ϕ*_*i *_(*i *= 1,..., 5) in Models 3 and 4 also contain zero which shows that the covariates are not significant factors in risk of preterm birth. This result shows that none of the variables make significant contributions to the explanation of the variation in risk. Recall that *η *= exp(*α*_*o*_) is a measure of the overall mean of the relative risk in the region under study. For each of the models, Table 3 gives the estimates of the overall risk together with its 95% confidence intervals. The overall mean of the relative risk is greater than 1 for each model which indicates that there is elevated risk of preterm birth across the whole of the Cape Breton municipality.

### Weighted regression results

The result of the fit is given in Table 4. None of the variables is significant in explaining the increased risk of preterm birth. The residual standard error for Model 4 was estimated to be 0.02347 on 137 degrees of freedom. Multiple *R*-square is 0.09795 which shows that the variables in the model account for about 10% of the total variation in the risk. The *F*-statistic for the regression relationship was estimated to be 2.479 on 6 and 137 degrees of freedom and the associated *p*-value is 0.0262. This shows that at least one of the parameters (*α*, and *ϕ*_*i*_) does not equal zero. Hence, there is evidence of a regression relationship between the dependent variable (*Y*_*i*_) and the area-specific variables (*z*_*i*_).

**Table 4:** Weighted regression result with parameter estimates, 95% CI, R-square, Residual standard error (RSE) and F-statistic (p-value)

Parameter	Model 2	Model 3	Model 4
*α*	-0.0996(-0.2513,0.0521)	-	-0.0878(-0.2364,0.0608)
*α*_*o*_	0.2325(0.1749,0.2901)	0.2092(-0.3219,0.7403)	0.2180(-0.3128,0.7488)
*ϕ*_1_	-	-0.0046(-0.0111,0.0019)	-0.0045(-0.0110,0.0020)
*ϕ*_2_	-	-0.0005(-0.0091,0.0081)	-0.0001(-0.0087,0.0085)
*ϕ*_3_	-	0.0111(-0.0073,0.0295)	0.0106(-0.0078,0.0290)
*ϕ*_4_	-	0.0026(-0.0107,0.0159)	0.0025(-0.0106,0.0156)
*ϕ*_5_	-	-0.0012(-0.0079,0.0055)	-0.0011(-0.0078,0.0056)
*R*^2^	0.0115	0.0891	0.0980
*RSE*	0.0241	0.0235	0.0235
*F*(*p – value*)	1.657(0.2002)	2.700(0.0232)	2.479(0.0262)

#### Test for autocorrelation

Next, Moran's I test was also carried out to examine whether there is spatial autocorrelation in the residuals. The result gave a correlation of -0.01628, variance of 0.002541 and standard error of 0.05041. In addition, the normal test statistic was -0.1843 with associated 2-sided *p*-value equal to 0.8538. These results are sufficient to conclude that there is no spatial autocorrelation in the residuals. Hence, there was no need to use spatial regression modelling.

## Discussion and conclusion

In practice, a typical spatial regression modelling will start with the examination of the dependent variable for spatial dependency. This can be done with Moran's I statistic or Geary C statistic. If there is no spatial pattern, then ordinary least squares or weighted least squares is sufficient to model the data. On the other hand if the dependent variable shows a spatial patterns, the first order spatial pattern can be incorporated at the beginning of the modelling using an adjacency matrix. However, great care has to be taken when using spatial modelling. First, some of the available parametric tests for measuring spatial autocorrelation, including Moran's I [37] and Geary's C [38] methods, are not robust when the data is sparse. The non-parametric rank-based method [39] is not available in most standard statistical software. Second, the structure of the adjacency matrix may affect the result. Hence, it must be chosen carefully. This research is part of a project done to assess the effect of maternal proximity to the hazardous waste from the Sydney Tar Pond, Nova Scotia. Two question have been addressed in this project: first, is maternal proximity to hazardous waste and pollution from the Sydney Tar Pond sites associated with increased risk of preterm birth? Second, how much of the variation in risk of preterm birth can be explained by socioeconomic inequalities across the study region?

In addressing these questions frequentist and Bayesian methods were employed. In the frequentist approach, Poisson regression for aggregated data and weighted least squares were fitted using distance from the Tar Pond and the following area specific-covariates: the proportion of persons who have no high school diploma; the rate of unemployment to population; the proportion of persons who are separated, divorced or widowed; the proportion of single parent families; and the proportion of people living alone. The same models were fitted using a Bayesian hierarchical model incorporating both structured and unstructured random effects to account for model overdispersion.

Our intention was to combine all of the area covariates to form the deprivation index, but income data were not available in 14 of the 144 enumeration districts included in the study. So the effect of each variable was assessed independently. The overall estimate of relative risk of preterm birth was found to be greater than 1 for almost all the enumeration districts. Also, none of the area covariates in the model is significant in explaining the risk of preterm births.

There was no evidence of any decrease in risk as we move away from the Tar Pond site. The results of both the weighted least squares and the quasi-likelihood Poisson regression agree with the result from the Bayesian hierarchical modelling which incorporates the spatial effects. The result of the Bayesian modelling shows that there is no significant spatial association of risk in the area studied. There was no obvious clustering of outcomes around the Tar Pond significant enough to find an association between maternal proximity to the Sydney Tar Ponds and risk of preterm birth. Although the three methods lead to similar results, we think the three-stage Bayesian hierarchical modelling is one of the best approaches for handling this problem. First, it allows the modelling of both sources of overdispersion, heterogeneity and spatial dependence or clustering in one model, and second, it allows the estimation of SIR with adjustment of sparse data. The least suggested method is the weighted least square method because it does not lend itself to some of the assumptions of Poisson models.

The following are some of the limitations of this research. First, data were not available for 14 of the Enumeration districts. Hence, they were omitted from our analysis but the effects of this on spatial dependency or our conclusion are not known. Second, we have based our analysis on the 1996 data but we do not have any evidence of whether the exposure from the Tar Pond has decreased since 1996. Third, the use of aggregated data may increase the potential for ecological bias which can occur due to the differences between individual and group-level estimates of disease risk. In particular, factors that affect length of gestation such as parity have not been directly adjusted for in the modelling.

Our experience with this project shows that more work is still needed in this area. None of the models was able to predict more that 10% of what we would like to know. The future plans include aggregating the data for up to ten years and modelling using other forms of *g*(*d*; *θ*). We will also consider using individual level data and incorporating other covariates. The study shows that there is an elevated risk of preterm births, which appears to be uniform across the whole of the Cape Breton regional municipality as shown by all the methods used. This shows that the pollution may be occurring on a wider scale and over time may have affected the ability to differentiate the EDs in terms of amount of exposure. A direct comparison of the Cape Breton regional municipality with other nearby municipalities may help answer some of the remaining questions.

## Competing interests

The author(s) declare that they have no competing interests.

## Authors' contributions

ASI and AC developed the project. All authors participated in preparing this manuscript.

## Pre-publication history

The pre-publication history for this paper can be accessed here:



## Supplementary Material

Additional file 1 **Bayesian diagnostic plots**. Gelman Rubin plots from five parallel chains, kernel density plots of sampled values for parameters of model 4 based on five pooled chains and autocorrelation plot for each chain. Click here for file
