# Supplementary material for: Comparison of Bayesian and frequentist approaches in modelling risk of preterm birth near the Sydney Tar Ponds, Nova Scotia, Canada
Source: BMC Med Res Methodol. 2007 Sep 10;7:39. doi: 10.1186/1471-2288-7-39 (PMC2080634; doi:10.1186/1471-2288-7-39)
Supplement: Additional file 1 — Bayesian diagnostic plots. Gelman Rubin plots from five parallel chains, kernel density plots of sampled values for parameters of model 4 based on five pooled chains and autocorrelation plot for each chain. [file 1471-2288-7-39-S1.pdf]

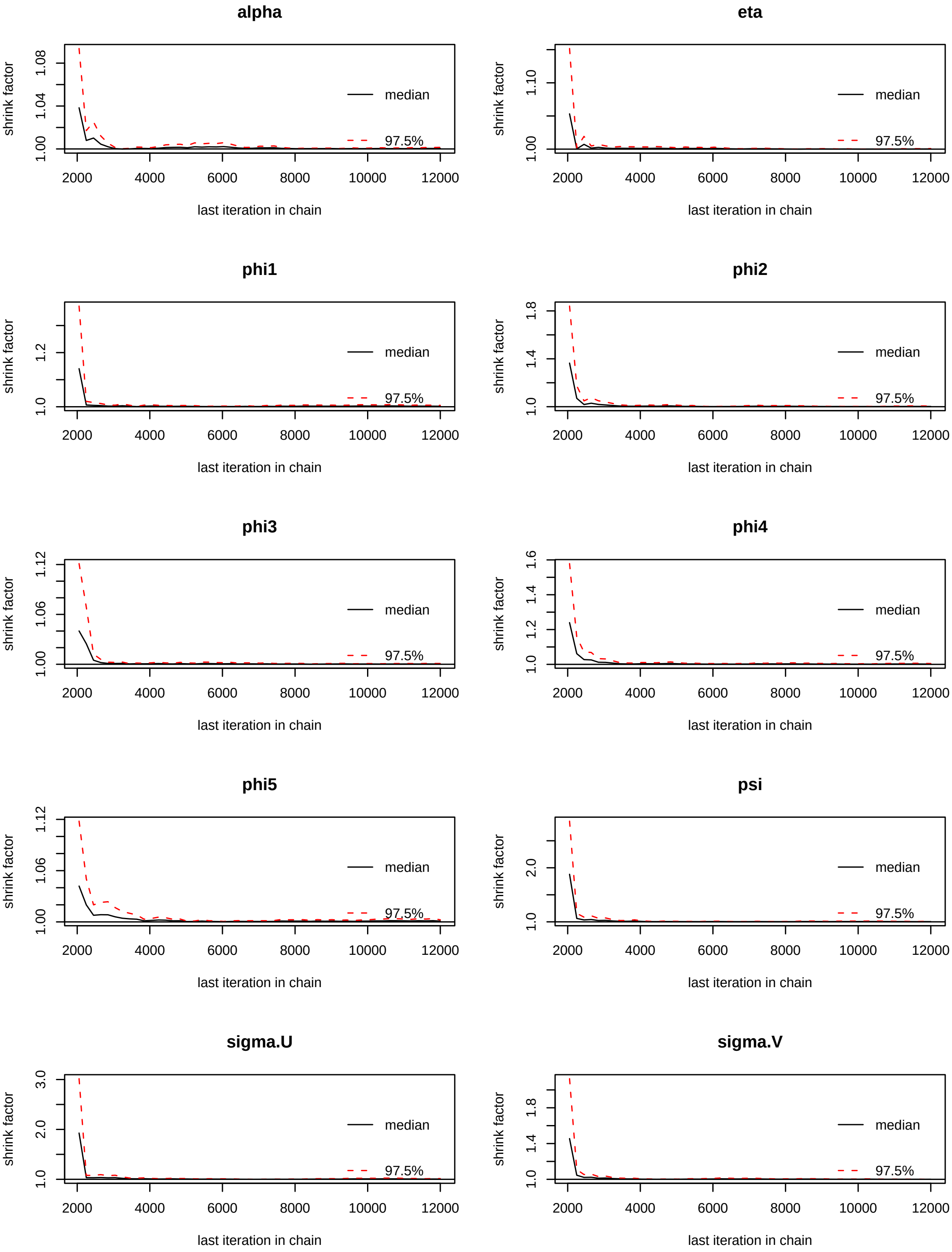

Figure 11: *Gelman Rubin plots from five parallel chains. Convergence is suggested when the medians and the 97.5 percentiles approach 1*

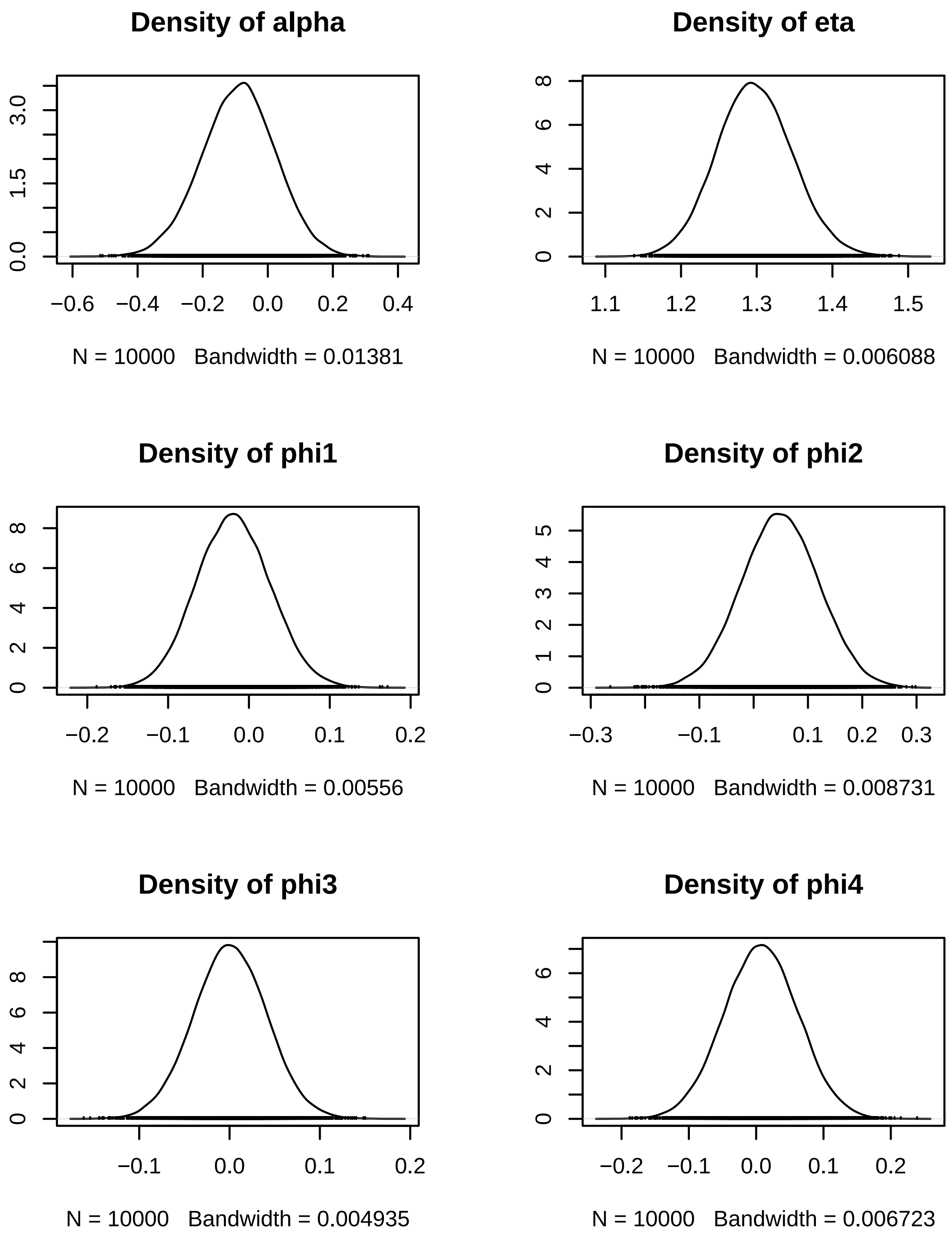

Figure 12: *Kernel density plots of sampled values for parameters of model 4 based on five pooled chains.*

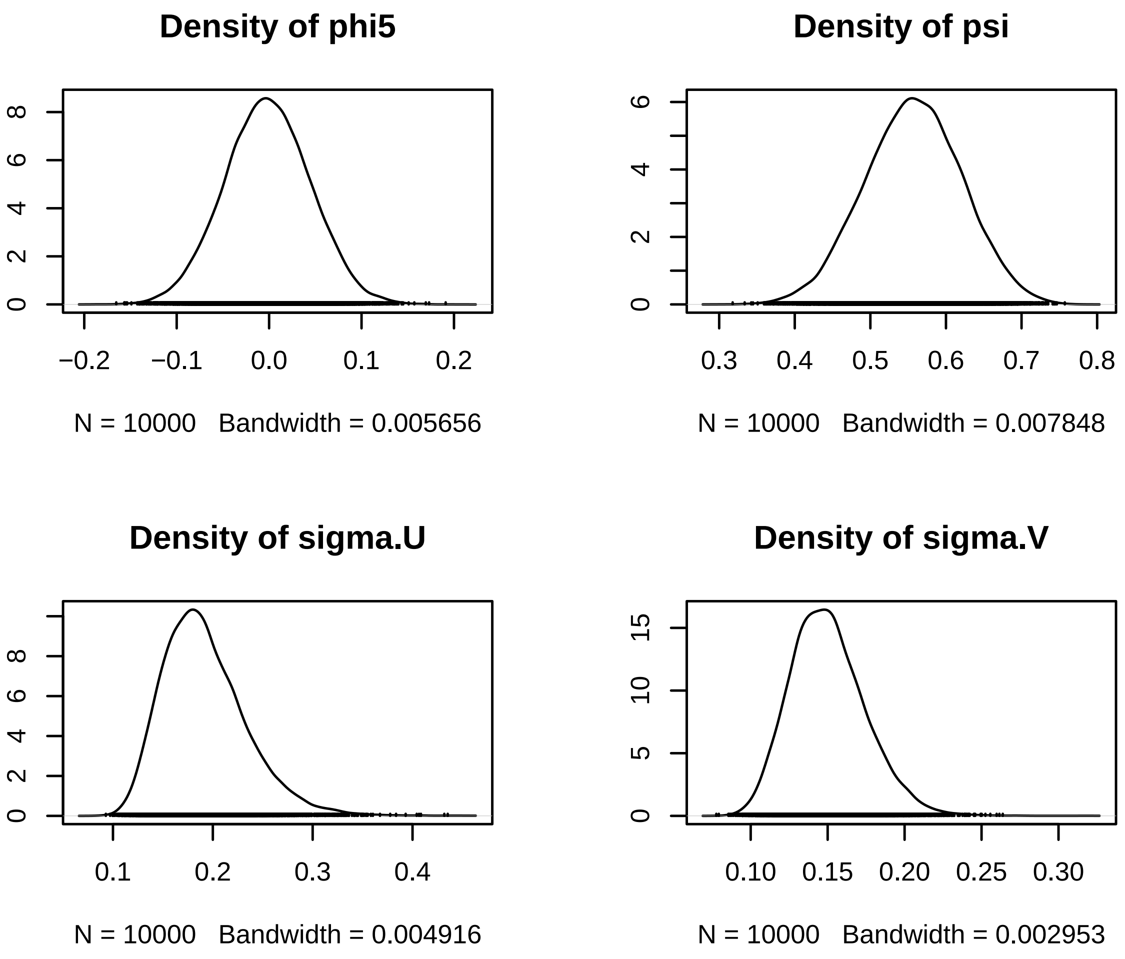

Figure 13: *Kernel density plots of sampled values for parameters of model 4 based on five pooled chains.*

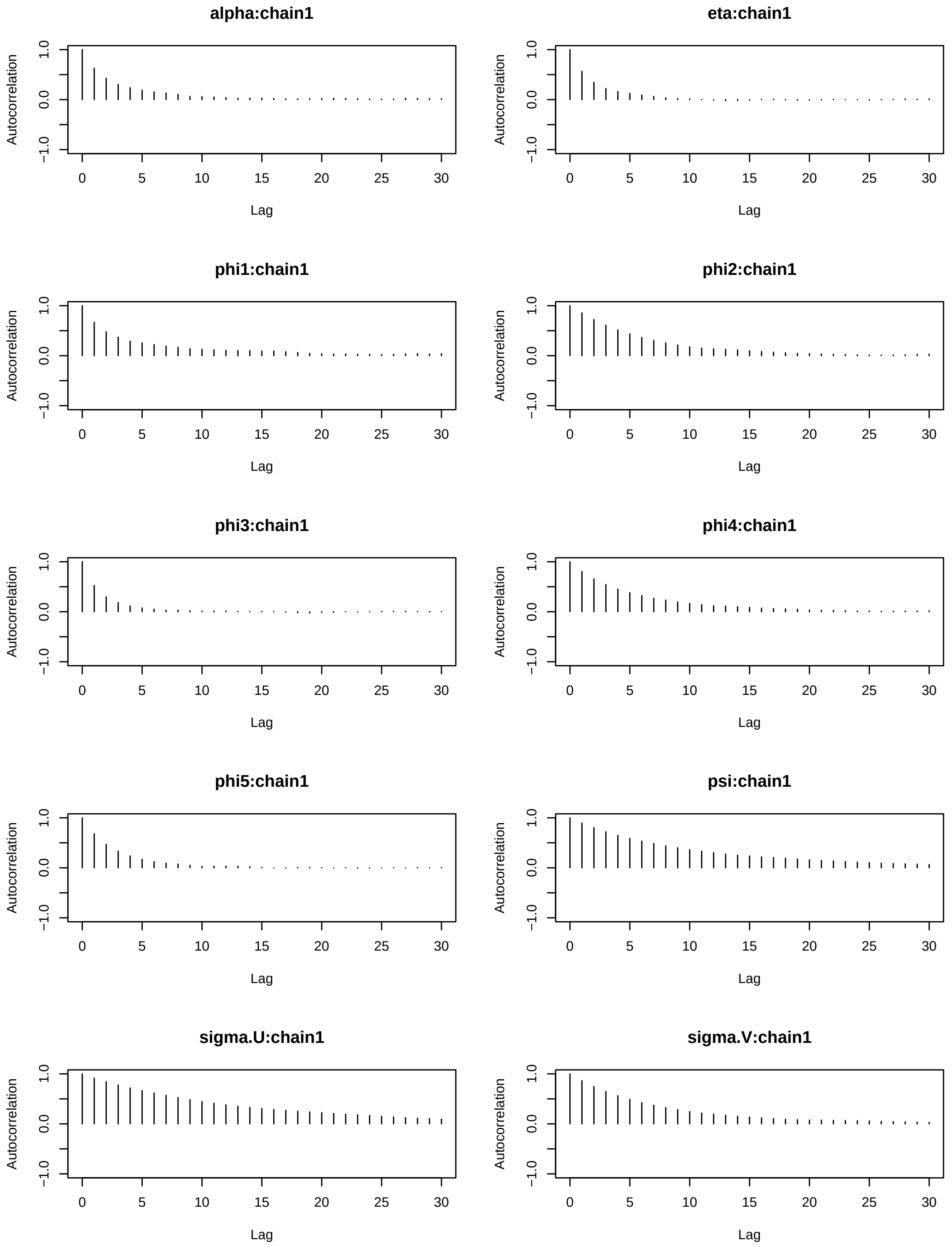

Figure 14: *Autocorrelation for chain 1*

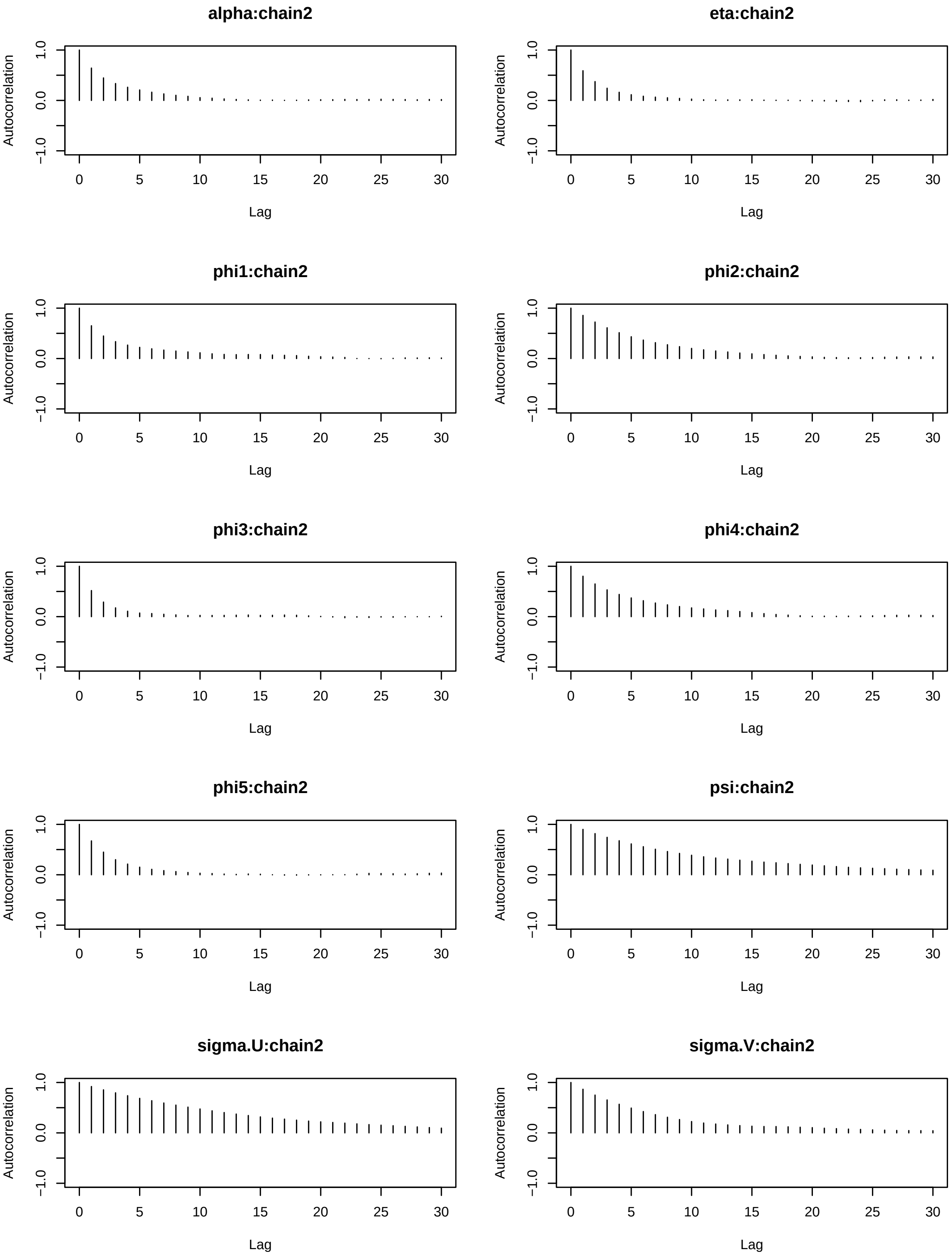

Figure 15: *Autocorrelation for chain 2*

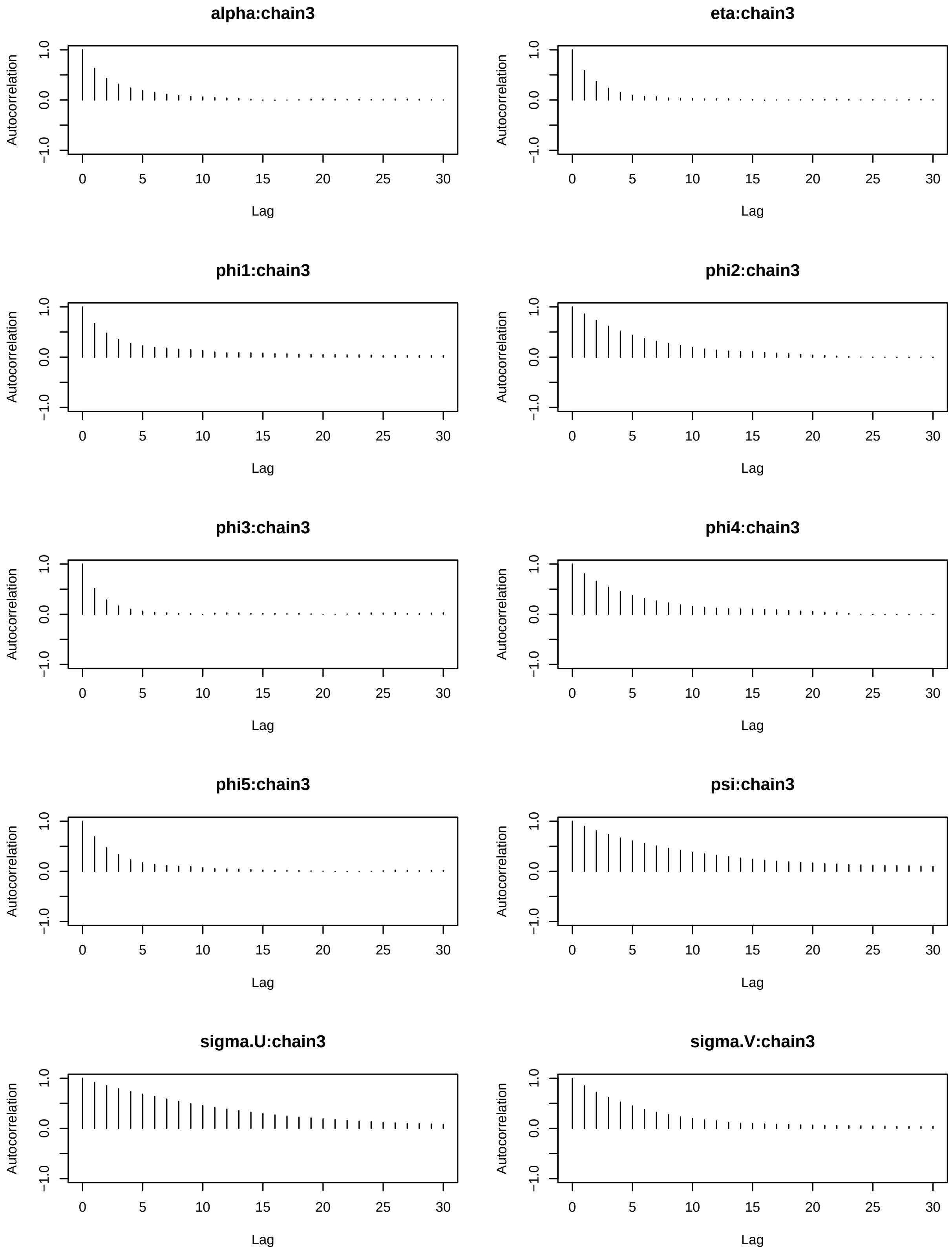

Figure 16: *Autocorrelation for chain 3*

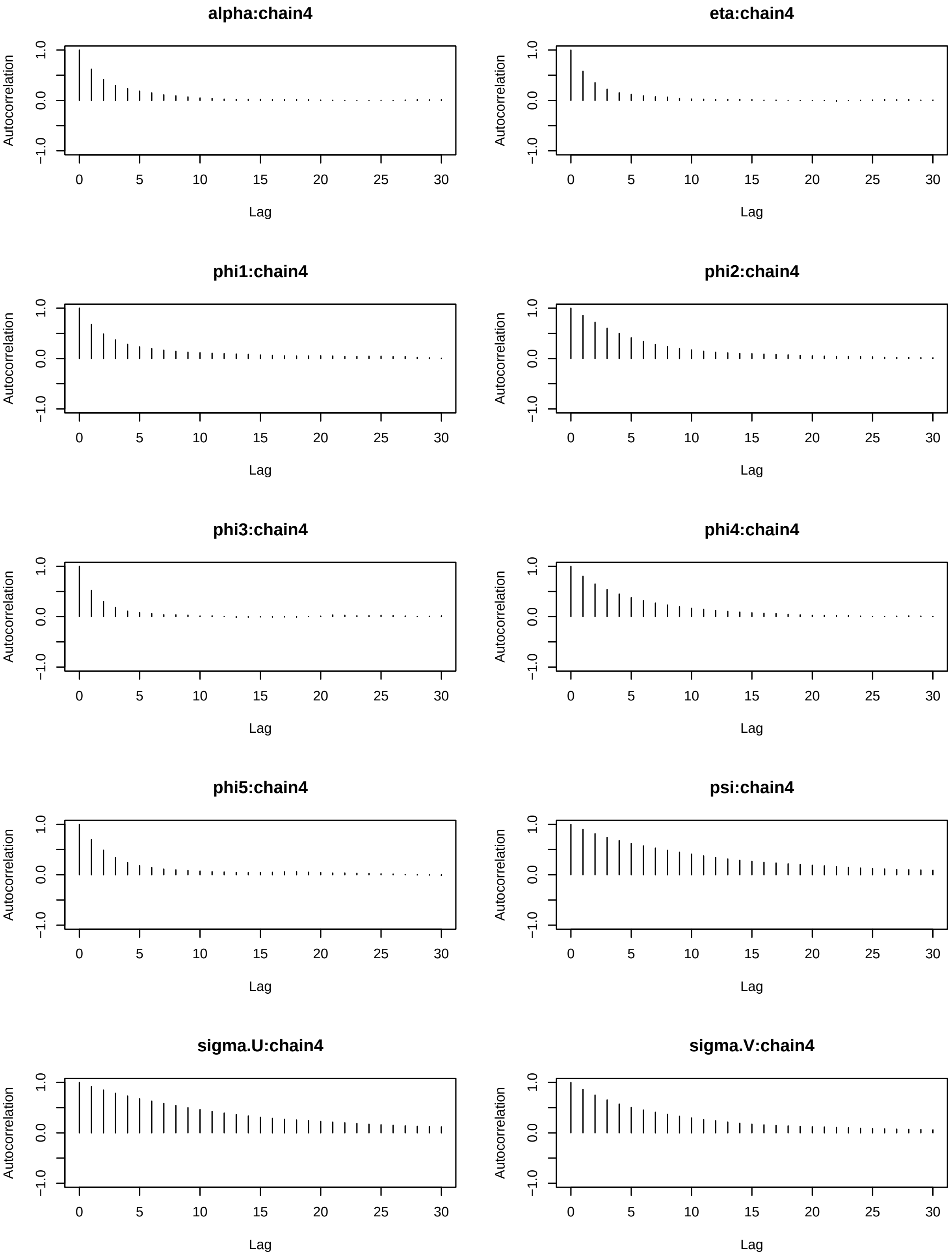

Figure 17: *Autocorrelation for chain 4*

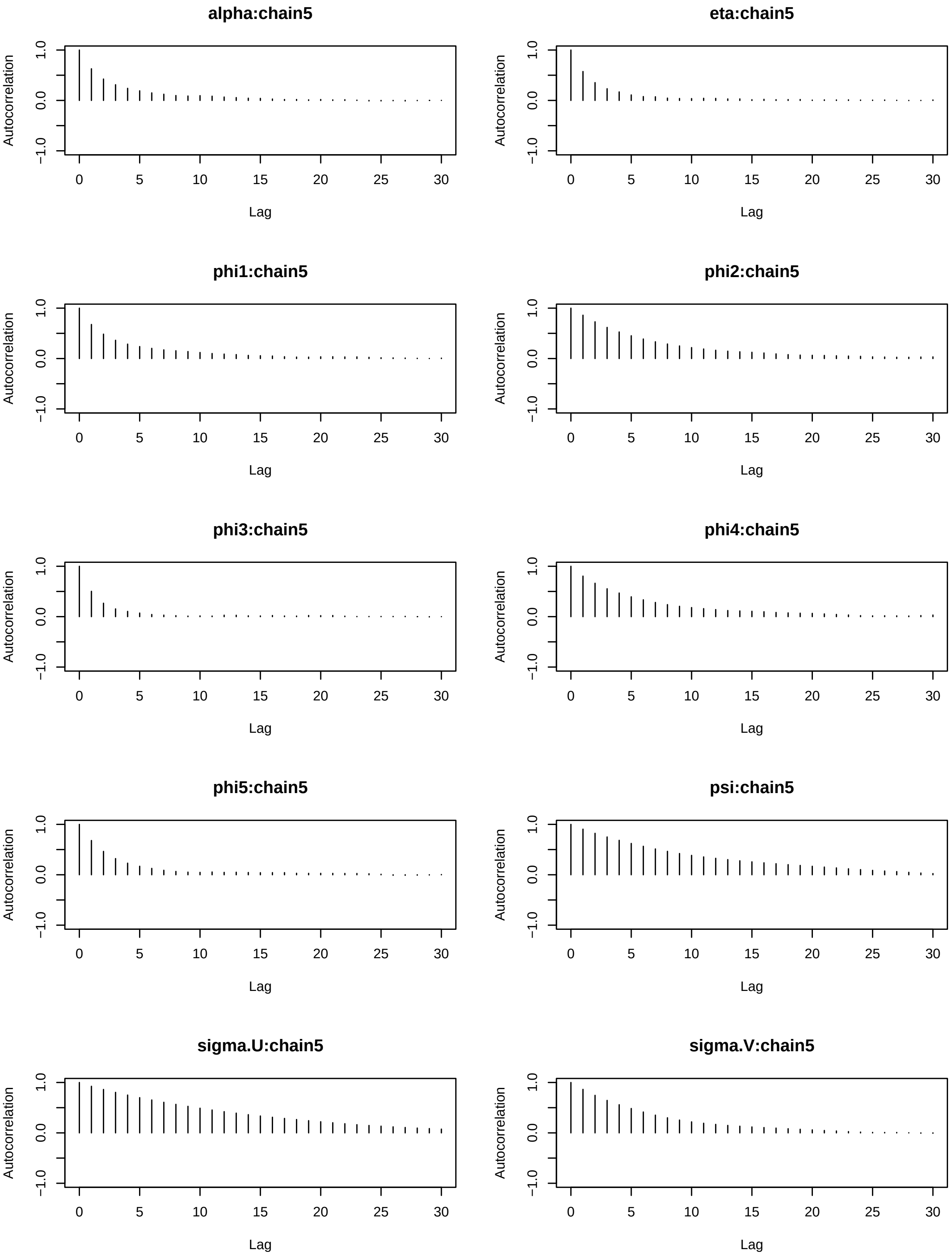

Figure 18: *Autocorrelation for chain 5*
